# Spatial Stereoresolution for Depth Corrugations May Be Set in Primary Visual Cortex

**DOI:** 10.1371/journal.pcbi.1002142

**Published:** 2011-08-18

**Authors:** Fredrik Allenmark, Jenny C. A. Read

**Affiliations:** Institute of Neuroscience, Newcastle University, Newcastle upon Tyne, United Kingdom; New York University, United States of America

## Abstract

Stereo “3D” depth perception requires the visual system to extract binocular disparities between the two eyes' images. Several current models of this process, based on the known physiology of primary visual cortex (V1), do this by computing a piecewise-frontoparallel local cross-correlation between the left and right eye's images. The size of the “window” within which detectors examine the local cross-correlation corresponds to the receptive field size of V1 neurons. This basic model has successfully captured many aspects of human depth perception. In particular, it accounts for the low human stereoresolution for sinusoidal depth corrugations, suggesting that the limit on stereoresolution may be set in primary visual cortex. An important feature of the model, reflecting a key property of V1 neurons, is that the initial disparity encoding is performed by detectors tuned to locally uniform patches of disparity. Such detectors respond better to square-wave depth corrugations, since these are locally flat, than to sinusoidal corrugations which are slanted almost everywhere. Consequently, for any given window size, current models predict better performance for square-wave disparity corrugations than for sine-wave corrugations at high amplitudes. We have recently shown that this prediction is not borne out: humans perform no better with square-wave than with sine-wave corrugations, even at high amplitudes. The failure of this prediction raised the question of whether stereoresolution may actually be set at later stages of cortical processing, perhaps involving neurons tuned to disparity slant or curvature. Here we extend the local cross-correlation model to include existing physiological and psychophysical evidence indicating that larger disparities are detected by neurons with larger receptive fields (a size/disparity correlation). We show that this simple modification succeeds in reconciling the model with human results, confirming that stereoresolution for disparity gratings may indeed be limited by the size of receptive fields in primary visual cortex.

## Author Summary

Stereo depth perception requires the brain to detect displacements of features between the two eyes' images. Several current models use local cross-correlation between the two eyes' images, looking for small patches that are the most similar between the two images. There is evidence that cells in primary visual cortex are doing something very similar. This model captures many aspects of human depth perception, notably why we can see depth variation on much coarser scales than luminance variation. This suggests that the spatial resolution for depth perception is set in primary visual cortex. However, the model as currently implemented cannot explain why humans are as good at detecting sine-waves in depth as they are at detecting square-waves, a fact that we have previously raised as a challenge to the model. Here we show that if we introduce a size/disparity correlation, such that larger patches are used when searching for larger displacements of features between the two images, then simple models based on local cross-correlation can explain human performance for both sine- and square-wave depth corrugations, without needing to invoke more complicated disparity processing. This supports the proposal that spatial resolution for depth perception is set in primary visual cortex.

## Introduction

**Figure 1:**
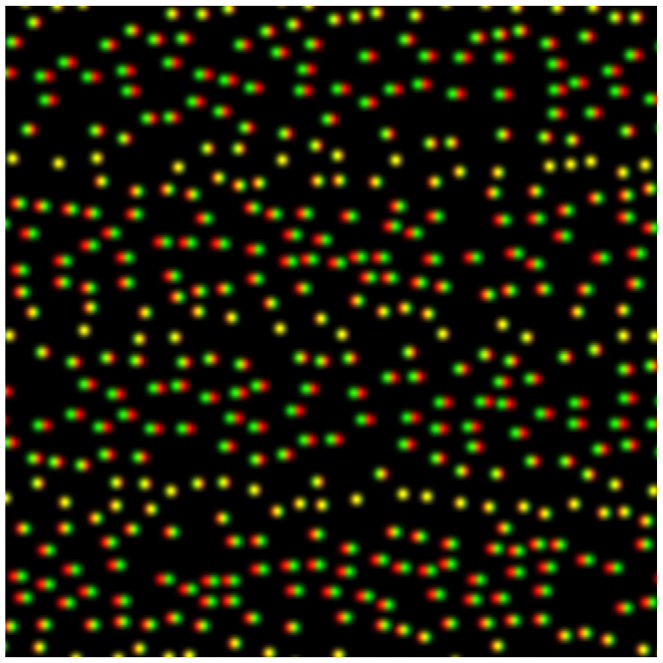
Sinusoidal disparity grating. Random dot stereogram of a sinusoidal disparity grating.

Human 3D depth perception is highly precise, with the ability to detect disparities between the two retinal images of less than the width of one photoreceptor [1]. However, it has very poor spatial resolution [2]–[4]. This can be demonstrated, for example, by using random-dot patterns to depict a corrugation in depth. An example is shown in Figure 1, depicted in red/green anaglyph stereo for illustration. The disparities between dots visible to the left eye (red) and right eye (green) vary sinusoidally as a function of vertical position in the image. Accordingly, when viewed with red/green 3D glasses, the dots appear to lie on an undulating surface rather like a sheet of corrugated iron, with the bars of the corrugations horizontal on the page. We shall refer to this kind of stimulus, pioneered by Tyler [3], as a sinusoidal disparity grating, by analogy with the luminance gratings pioneered by Schade [5].

The upper frequency limit at which such disparity gratings can be perceived has been found to be around 3–4 cycles per degree [3], [6]–[8] which is much lower than the limit found for luminance gratings. This low spatial stereoresolution has been explained in terms of a model, based on the known properties of cells in primary visual cortex (V1), where disparity is measured by the use of local cross-correlation between the two eyes' images [6], [8], [9]. Banks et al. found that the spatial stereoresolution of the model depended on the size of the correlation window, roughly corresponding to the receptive field size of the V1 cells modelled, such that the resolution was higher for smaller windows, up to a limit set by the highest useful dot density which in turn depends on the level of optical blur [6], [8]. For realistic levels of blur, they found that the smallest useful window size was roughly 6 arcmin. They also found that the performance of the model with this window size showed the most similar dependence on the level of blur to that of human observers, suggesting that “the smallest mechanism in humans has a diameter of roughly 3–6 arcmin, which is the smallest useful size given the optics of the human eye” [8]. Based on the success of this model, these authors made the interesting and plausible suggestion that spatial stereoresolution may be set in primary visual cortex, reflecting the size of receptive fields there [6], [9]. In this view, the better spatial resolution for luminance gratings occurs because V1 receptive fields are divided into ON and OFF subregions; the effective window reflects the size of V1 subregions. Because V1 neurons respond best to locally uniform disparity [9], the effective window for disparity is the entire receptive field.

**Figure 2:**
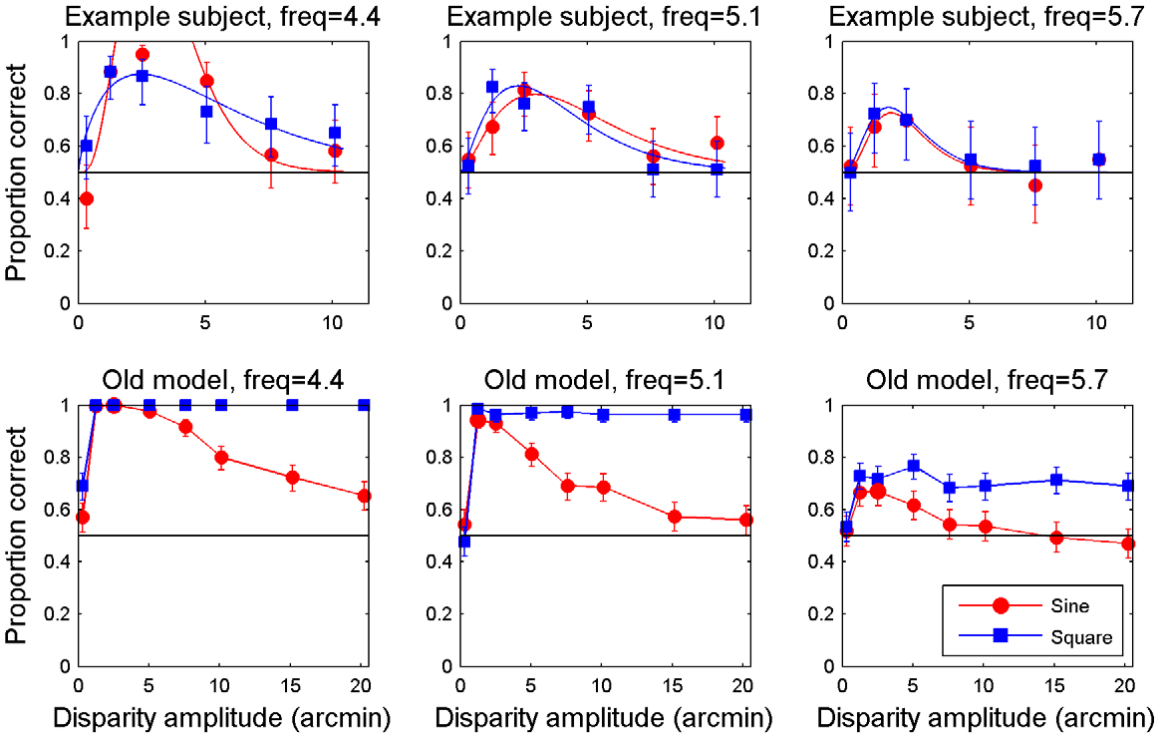
Comparison between human data and model results with the old fixed window-size model. Examples of human data (top row) and model results (bottom row) reproduced from Allenmark & Read (2010). The task was to detect which of two intervals contained a disparity corrugation, and which contained disparity noise with the same disparity amplitude. The model is the old fixed-window-size cross-correlation model with the decision model based on template matching.

This is an intriguing and attractive model, which relates human perception to the properties of neurons early in visual cortex. However, we recently raised an observation which potentially presents a challenge to this view [10]. Almost all previous empirical results relating to stereo resolution were obtained using sine-wave disparity gratings like that depicted in Figure 1. Because the model uses detectors which are tuned to locally uniform patches of disparity it would be expected to perform better on detection of square-wave gratings, which consist of regions of locally constant disparity. We recently confirmed this with simulations using the model of Banks et al [6], [8] with the optimal window size of 6 arcmin. As expected, the model does indeed perform better with square-wave gratings, in particular at high disparity amplitudes. However, Tyler [4] had found using line stereograms that performance was similar in both square- and sine-wave disparity gratings. We therefore tested human observers on dense random-dot stereograms depicting square-wave gratings. We found that the model's prediction was not borne out: humans never showed significantly better ability to detect square-wave than sine-wave gratings [10]. Figure 2 shows example human and model data near the upper frequency limit, illustrating the marked qualitative difference between the model and the human observers. For humans (Figure 2, top row), performance rises rapidly to a peak and thereafter declines as the grating's disparity amplitude increases, for both sine-wave gratings (red circles) and square-waves (blue squares). The model (bottom row) performs similarly for sine-waves, but for square-waves, the model's performance remains at its peak value as disparity amplitude increases, in disagreement with the human data.

This failure of the model raises the possibility that spatial stereoresolution may not be limited by the smallest receptive field size in V1 after all but rather at a later stage, perhaps by detectors in extra-striate areas tuned to disparity slant or curvature [11]–[14]. However, there is also the possibility that minor modifications to the model may make it consistent with these new human results.

In this paper, we examine a modified version of the model, where larger disparities are detected using larger correlation windows. There is considerable psychophysical evidence for such a size/disparity correlation [2]–[4], [15]–[17], and some physiological evidence has also been found in favour of it [18]. We show that this new version can capture human performance on both sine- and square-wave depth corrugations.

## Methods

### Model

#### Stimuli and task

The stimuli and task have been described in detail elsewhere [10]. Briefly, the stimuli used were random-dot stereograms depicting horizontal sine-wave and square-wave disparity gratings (i.e. modulations in disparity as a function of vertical position in the image). For humans, the disparity gratings are readily visible at low grating frequencies, but as the frequency increases, it becomes impossible to detect the distinct bars of the corrugation, and the dots either appear to be distributed throughout the space between the front and back limits of the stereogram or they appear to be distributed over two planes at the front and back limits, depending on the waveform and amplitude of the grating. Disparity gratings at frequencies beyond the limit of stereoresolution thus remain readily distinguishable from planes of constant disparity or from binocularly uncorrelated dot patterns, but the surface structure cannot be perceived. Accordingly, to probe stereoresolution, we asked subjects to distinguish disparity gratings from disparity noise patterns containing the same range of disparities. Each trial consisted of two intervals. Observers were shown one stereogram depicting a sine- or square-wave grating and one stereogram of the corresponding noise pattern, and had to judge which stereogram contained the grating.

In the psychophysics experiments, sine- and square-wave gratings were interleaved so that human observers did not know which sort of grating to look for on any given trial. Disparity grating amplitude and phase were also randomly interleaved, but different frequencies were run in blocks. The computer simulations reflected the human experiments as closely as possible, so the model observer had no prior knowledge of grating waveform, amplitude or phase. The images presented to the model were preprocessed by blurring and scaling to simulate the optics of the human eye, as in the model of Banks et al. [6], [8] and our previous paper [10].

#### Encoding disparity using cross-correlation

After the preprocessing, the images were presented to a population of cross-correlators tuned to different vertical locations along the grating and to different disparities between left and right eyes. Each cross-correlator had two windows, one in each eye's image. Both windows for a given cross-correlator had the same vertical position. In our model, the left-eye window was always at the same horizontal position. The right-eye window was in one of a range of horizontal positions on either side of the left-eye window. The correlation between contents of the two windows was calculated and recorded for every combination of window-positions. The definition of correlation that was used was:
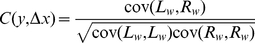
(1)where 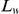 and 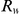 are the pixel-values in the left and the right image, multiplied by the window function, and cov is the covariance. We used Gaussian window functions that were cut off at two standard deviations from the centre. That is, if the left window is centered on position (x,y) and I_L_(i,j) represents the left eye's image at position (i,j), then

L_w_ is the set of values 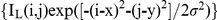 for all (i,j) satisfying |i-x|<2σ and |j-y|<2σ, and

R_w_ is the set of values 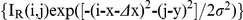 for all (i,j) satisfying |i-x|<2σ and |j-y|<2σ.

We refer to the standard deviation σ as the size of the window for that cross-correlator. The function C(y,Δx) represents a population of neuronal units tuned to different disparities Δx and vertical image positions y. The preferred disparities used were in the range from −13 to 13 arcmin with a step of 0.6 arcmin (1 pixel in the scaled images), except in the section on “Size-disparity correlation and the disparity gradient limit”, where we included window disparities up to 140 arcmin, again with a step size of 0.6 arcmin, in order to examine performance down to lower frequencies. The step size in the range of y-positions was also 1 pixel in the scaled images.

The innovative feature of the present paper is that cross-correlators tuned to larger disparities, i.e. with larger separations between the centers of their left-eye and right-eye windows, had larger windows. Psychophysical evidence for a different sort of size-disparity correlation was provided by Smallman and MacLeod [16].These authors investigated the optimal disparity at which subjects could perform a front back discrimination task with stereograms based on narrow-band filtered noise. They obtained linear fits between optimal disparity and the center spatial frequency of the noise on a loglog scale. Assuming that cells processing higher luminance frequencies have smaller receptive fields, this provides evidence for a correlation between disparity tuning and receptive field size. The fits obtained for the data from the two different subjects tested had loglog slopes of approximately −1 and −0.5, corresponding respectively to a linear and a quadratic relationship between size and disparity. Motivated by Smallman and MacLeod's results, we have examined a second order polynomial as well as a linear function as the relationships between window size and preferred disparity in our model:
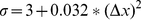
(2)

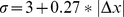
(3)where σ is the standard deviation of the Gaussian window and 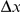 is the disparity of the window, both measured in arcmin. We have also explored an exponential size disparity relationship. Although the very long run-time of the simulations made it impossible to perform systematic optimization, or to fit the model results to the data of individual subjects, the size/disparity relationships given in Equation 2 and Equation 3 gave the best match to human performance of those we examined.

**Figure 3:**
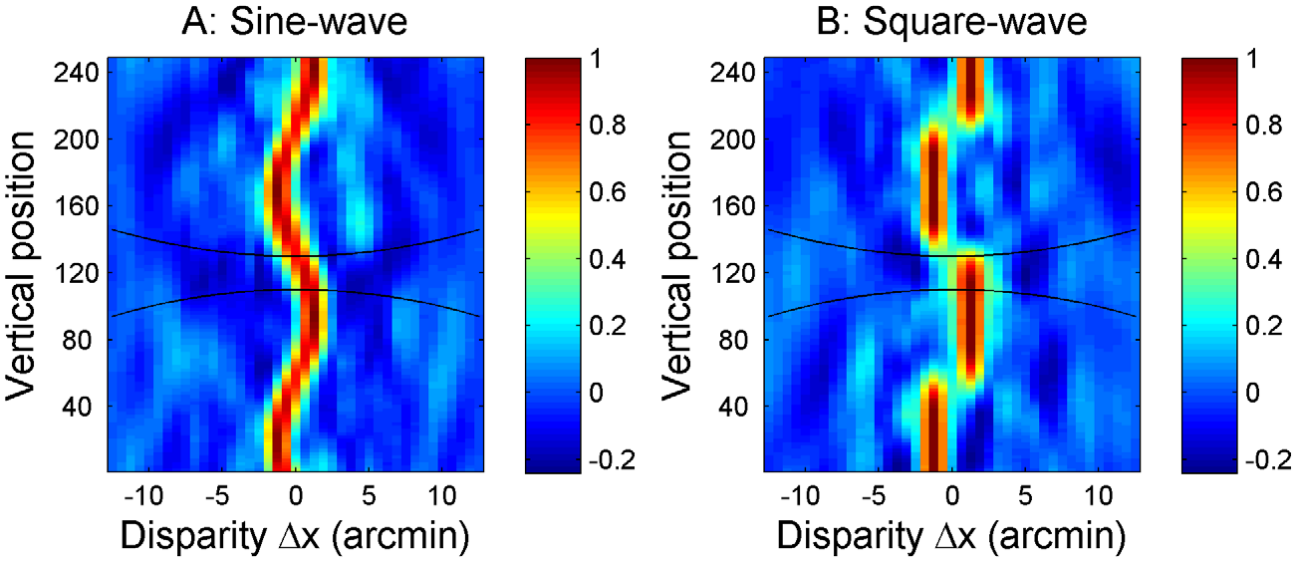
Examples of correlator output. Legend: Examples of output from the cross-correlator for one sine-wave and one square-wave disparity grating, both with a frequency of 1.3 cpd. A Gaussian window with σ = 3+0.032*(Δx)^2^ arcmin was used. The black lines shows the extent of the correlation window, taken to be the 1SD contour of the Gaussian.

The cross-correlator output can be visualised as a two-dimensional image showing correlation as a function of the horizontal disparity, Δx, between the windows as well as the vertical position of the windows, y (see Figure 3). This cross-correlation performs the initial encoding of disparity within the model. Physiologically, we envisage this as occurring in primary visual cortex. The cross-correlation calculated for a given window position, size and disparity represents, in idealised form, the combined activity of several disparity-selective neurons in primary visual cortex, all tuned to the same retinal position and disparity. Each row in Figure 3 represents the activity of a group of V1 neurons tuned to the same retinal location but to a range of horizontal disparities. The black lines indicate how the vertical extent of the window increases with the horizontal disparity to which they are tuned.

#### Making a perceptual judgment

In order to compare our model to human observers, we needed to take the correlator output from each interval, and use it to make a judgment regarding which interval contained the grating. Physiologically, this process presumably occurs in extra-striate areas, but little is known about how it is achieved. We therefore have little to go on in modelling this process other than some plausible assumptions. In this paper, we shall ultimately conclude that spatial stereoresolution is fundamentally limited by the initial encoding of disparity in V1, not by the nature of this perceptual read-out process. It is therefore important to demonstrate that our results are qualitatively the same independent of the precise assumptions made regarding read-out. To this end, we have examined three different decision models incorporating specific decision rules, aiming to span a range of possible approaches and assumptions. Since these all give qualitatively similar results, we present only one of them in the main body of the paper. The others are presented in Text S1 and Text S2.

For the results in the main body of the paper, we assume that the model observer knows the frequency of the grating it is trying to detect, though not the disparity amplitude, waveform (sine vs square) or phase. This is realistic since frequency was blocked in the psychophysical experiments whose results we are trying to reproduce, while amplitude, waveform and phase were interleaved. Avoiding the need to search for frequency speeds up the simulations, but is not critical to our results. In Text S1, we show that very similar results are produced by a model which does not know frequency.

This method used a set of templates of the correlator output, representing the brain's prior knowledge of the average V1 activity caused by different stimuli. This is closely based on the approach taken by Tsai & Victor [19]. We assume that the brain knows (or is able to reconstruct) the activity expected in response to all the different stimuli used in our experiment, both gratings and noise, based on prior experience. This assumption is discussed further in the Discussion.

**Figure 4:**
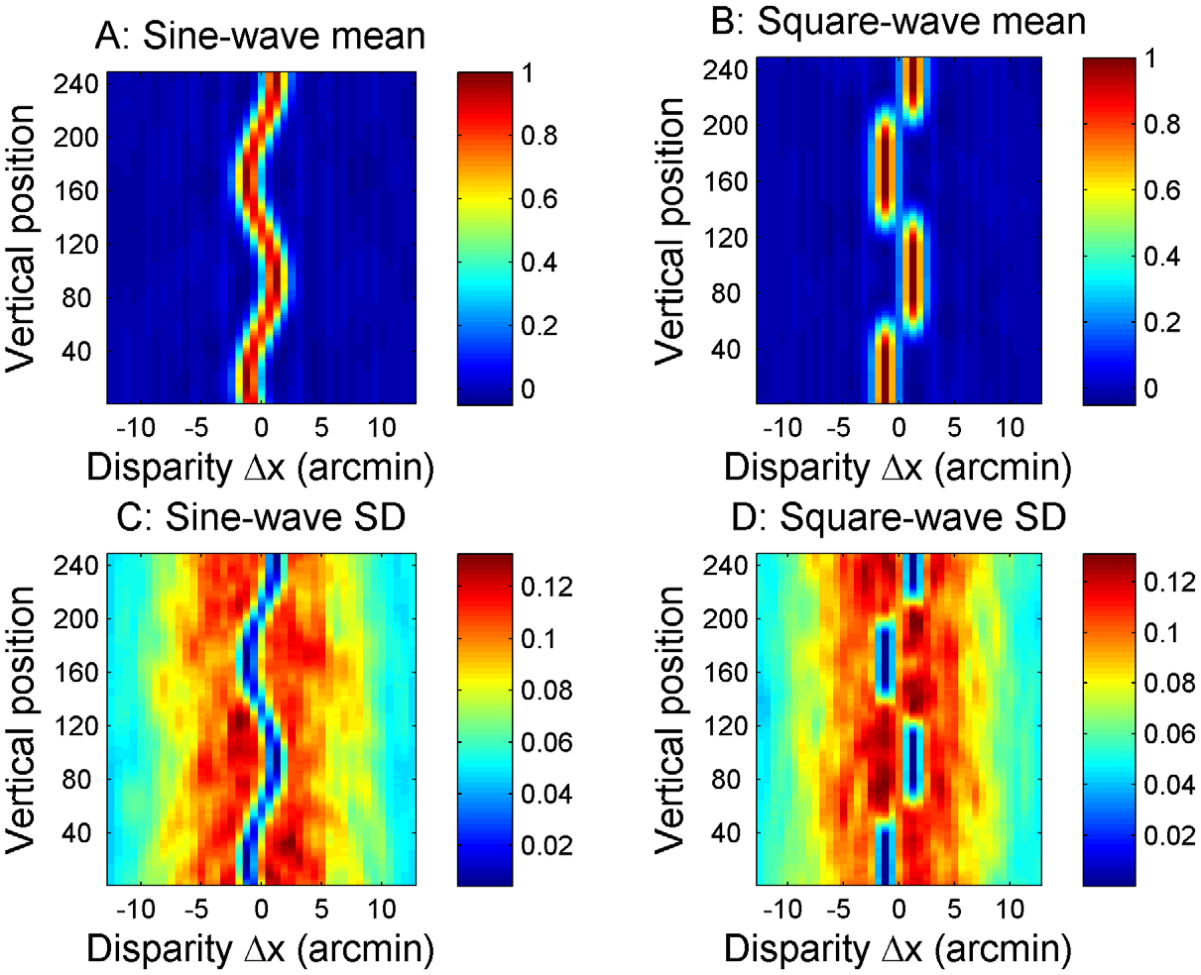
Examples of grating templates. Examples of templates for sine-waves (left) and square-waves (right) with a frequency of 1.3 cpd. The upper row shows the mean and the lower row shows the standard deviation for cross-correlators tuned to vertical position y and disparity Δx, estimated from 100 different random-dot disparity gratings. Figure 3 showed analogous results for a single grating.

The template for each type of stimulus was generated by making 100 different random dot stereograms, preprocessing them with the same preprocessing steps that were used in the main model, and then passing them to the cross-correlator. The mean and standard deviation for each position y and disparity Δx were then calculated based on the resulting set of 100 correlation images (see Figure 4). This process was repeated for gratings of different frequencies, amplitudes, phases and waveforms (sine vs square). The phase of the disparity gratings was varied in steps of 10°. When testing the model, the phase was randomly chosen at each trial to be one of the 36 different phases represented in the set of templates. The disparity amplitudes were 0.3, 1.3, 2.5, 5.1, 7.6, and 10.1 arcmin. Thus there were 432 grating templates per frequency, reflecting 36 phases×6 amplitudes×2 grating waveforms. Noise templates were by their nature independent of frequency and phase, so there were 12 noise templates in total, reflecting 6 amplitudes×2 waveforms.

To simulate an experiment, we assumed that the frequency was known, so the model was using the 432 grating templates for the correct stimulus frequency, as well as the 12 noise templates. In each interval, the correlator output from this stimulus was compared to each of the 432 grating templates, by calculating the Pearson correlation coefficient between the current and each different grating template [20]. The quality of the match to the best-fitting grating was taken to be
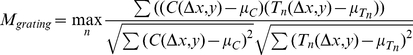
(4)where *C* is the correlator output, 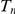 is the n^th_^ grating template, 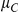 and 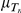 are the means over all disparities 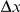 and all y-positions of the correlator output and template 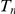 respectively, and the sums are over all 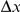 and all y. The maximum is taken over all values of n, from 1 to 432.

We then calculated the difference (M_grating_-M_noise_) for each interval, and judged the grating to be in the interval for which this difference was greater.

## Results

### Cross-correlation can be obtained from energy-model units

The cross-correlation coefficient used in the present paper as well as by Banks et al. differs in a number of ways from the cross-correlation implemented by the energy model. First, it is normalized to lie between 1 (for perfect interocular correlation) and −1 (for anti-correlated stimuli). Second, it operates on the retinal images directly, not the images after filtering by a bandpass receptive field. Finally, the multiplication of the two images is performed first, followed by integration over space, unlike the energy model where the images are integrated over space first and the results are then multiplied together. This has the consequence that the cross-correlation model used here depends more critically on the exact relative positioning of visual features in the two images compared to an energy model unit of the same window-size, and that its disparity tuning is finer and independent of window size. Given that we are claiming our results show that disparity resolution is limited by activity in primary visual cortex, it is important to be clear how the idealized cross-correlation computed in our model relates to more realistic models of individual neurons.

To this end, we begin the Results section by showing that the output of a Banks-style cross-correlator can be approximated by suitably combining the responses of many complex cells tuned to different orientations and frequencies.

In the standard energy model the response of a stereo energy unit is described by the equation:
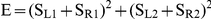
where
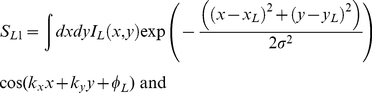


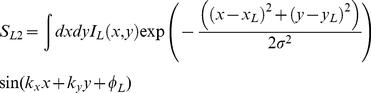
and I_L_ is the left eye's image, the wavenumbers k_x_ and k_y_ together specify the spatial frequency and orientation of the cells receptive field, x_L_ and y_L_ specify the position of the center of the left eye's receptive field, φ_L_ is the phase of the receptive field, and σ is the standard deviation of the Gaussian envelope of the receptive field. S_R1_ and S_R2_ are defined analogously. We assume that, due to adaptation at lower levels of the visual system, the image is defined relative to the overall mean luminance, so that averaged across the whole image, 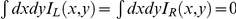.

Let us assume there are also monocular complex cells which compute
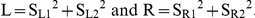
The response of the energy model unit can be split into a binocular part B and monocular parts L and R:
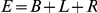
where
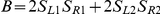
Now we compute the total response of all cells at this location which have phase disparity zero and position disparity Δx, summing over cells tuned to a range of spatial frequencies and orientations. In Text S3, we show that integrating B in this way over all spatial frequencies and orientations gives us
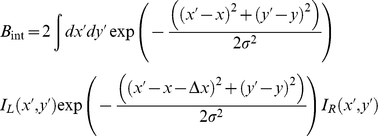
Approximating the integrals with a sum over pixels, and using L_W_ to represent the image after multiplication by the window function, this is
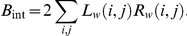
This is simply the covariance of the weighted image-patches, plus a term reflecting the average pixel-value within the window:
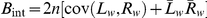
where *n* is the total number of pixels included in the sum. Similarly, integrating the monocular terms over all spatial frequencies and orientations, we obtain
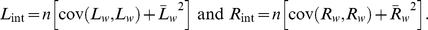
Now we use the monocular terms to normalise the binocular term [19]–[21]:
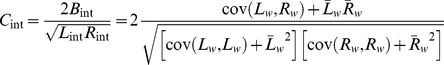
The normalisation ensures that C_int_ remains between +1 (for units tuned to the stimulus disparity, where L_w_ = R_w_) and −1 (for anti-correlated stimuli, where L_w_ = −R_w_).

For random-dot patterns where the correlation window is large compared to the dot-size, the average pixel-value within each eye's window will be very nearly the same as the average pixel-value across the whole eye's image, which is zero by definition. For such images, C_int_ reduces immediately to C as defined in Equation 1. For natural scenes or other images where the luminance undergoes large-scale changes across the image, this would not be the case, and C_int_ would not be zero for binocularly uncorrelated images. Real neurons have not been studied with such images, so it is not possible to say whether C_int_ or C as defined in Equation 1 would be more appropriate in that case.

This analysis shows that the key features of the Banks model – units sensitive to the precise location of features within the window, isotropic windows, disparity tuning curves whose width is independent of window size – can be produced within a more physiologically-realistic model, simply by combining the outputs of energy-model units tuned to many spatial frequencies and orientations. Essentially, the Banks model is a computational short-cut which enables us to approximate the properties of a much larger population of energy-model units at vastly reduced computational cost. This is somewhat analogous to how the energy-model itself uses a quadrature pair of units with 0 and π/2 phase to approximate the output of a large number of subunits tuned to a range of phases. This derivation gives us confidence that the encoding stage of our model, while clearly highly idealised, is nevertheless consistent with the physiology of early visual cortex.

We now move on to examine how the model performs when its outputs are used to perform our psychophysical task, under various different decision models.

### Size-disparity correlation makes sine- and square-wave gratings equally detectable

**Figure 5:**
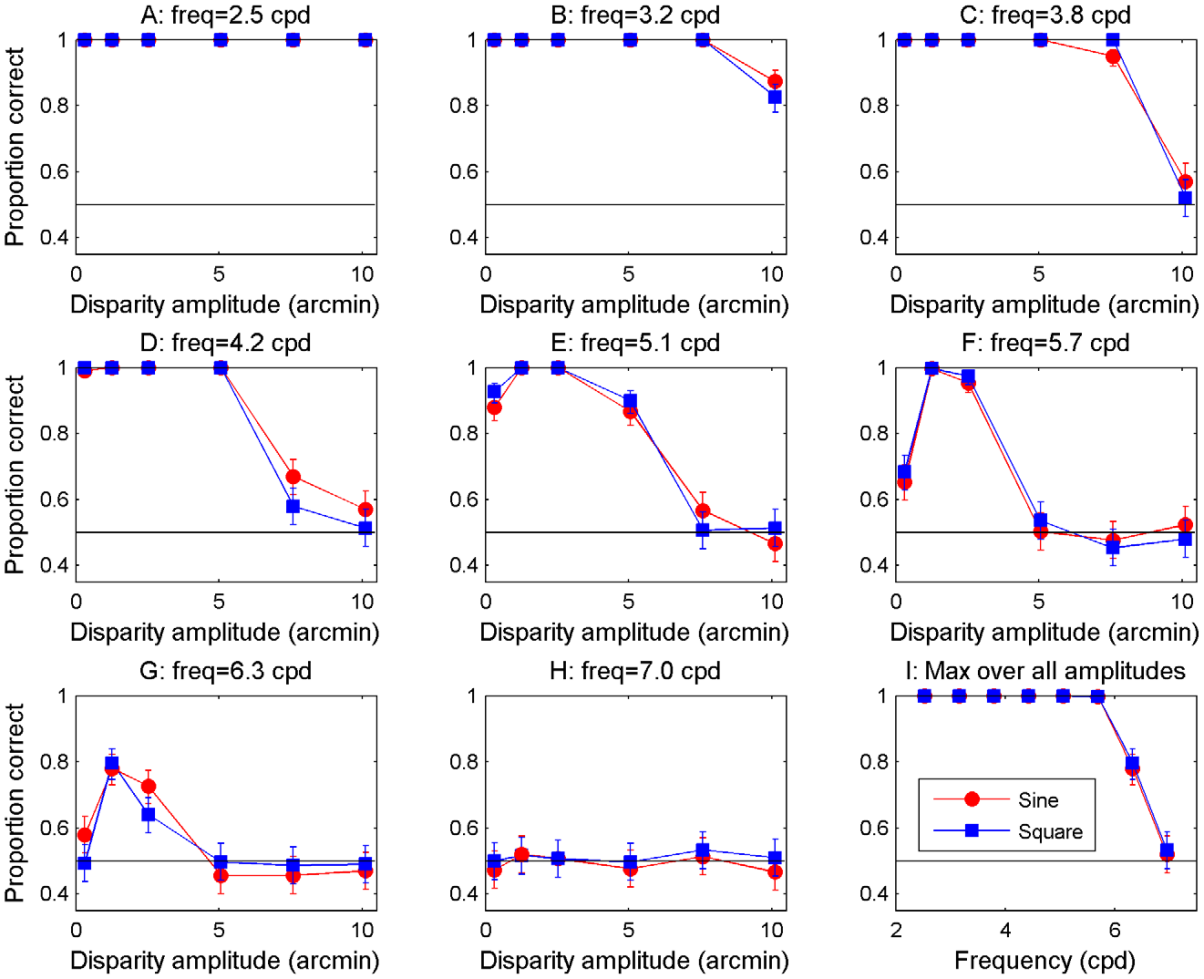
Model results with the quadratic size-disparity correlation. Legend: Model performance on the grating detection task as a function of amplitude and frequency. The last plot (I) shows the maximum performance over all amplitudes for each frequency. This is for the model with the template matching decision model with known frequency and a quadratic size-disparity relationship (Equation 2).

Figure 5 shows the results of the model. Panels A–H show the model's performance (percent correct judgments) as a function of disparity amplitude for different grating frequencies and the final panel shows the maximum performance, i.e. that at the optimal disparity amplitude for each frequency, as a function of frequency. Red circles show results for sine-wave gratings; blue squares those for square-wave gratings. Throughout, error bars show 95% confidence intervals. Critically, the results are now very similar for both sine- and square-wave disparity gratings – like human observers and unlike the original model (Figure 2). Like human observers, as disparity amplitude increases beyond its optimal value, performance for both grating waveforms decays back to chance.

Similar figures are given in Text S1 and Text S2 for alternative decision models (Figure S1-1 in Text S1 and Figure S2-2 in Text S2). Unsurprisingly, there are quantitative differences between the results from different decision models, especially in the percent correct at the lowest disparity amplitude. This amplitude, 0.3 arcmin, is below the step size of 0.6 arcmin in the range of correlation detectors, and the decision models vary in how efficient they are at extracting information at this sub-step-size disparity. Similarly, the decision models vary somewhat in the frequency at which peak performance first starts to decline. We know in principle how to match human performance on both of these. Capturing sensitivity to small disparity amplitudes would require the right minimum spacing in the population of cross-correlators, plus the addition of noise to limit the ability to discriminate tiny disparities. Capturing the correct frequency at which performance declines would require us to tweak the minimum window-size, i.e. the value of the first term in Equation 3, as done by Banks et al [6], [8]. Given the long simulation run-time and the fact that these issues are solved in principle, we have not here attempted to chase down these parameters further.

In Figure S2-2 in Text S2, showing results for a decision model based on auto-correlation, there are a couple of frequencies where performance starts dropping for the sine-waves at slightly lower amplitudes than for the square-waves. Interestingly, 2 of our 4 human observers also displayed this tendency (Figure 10 of [10]), while neither humans nor model ever displayed an earlier drop for square-waves than for sine-waves.

### Form of the size-disparity correlation is not critical

**Figure 6:**
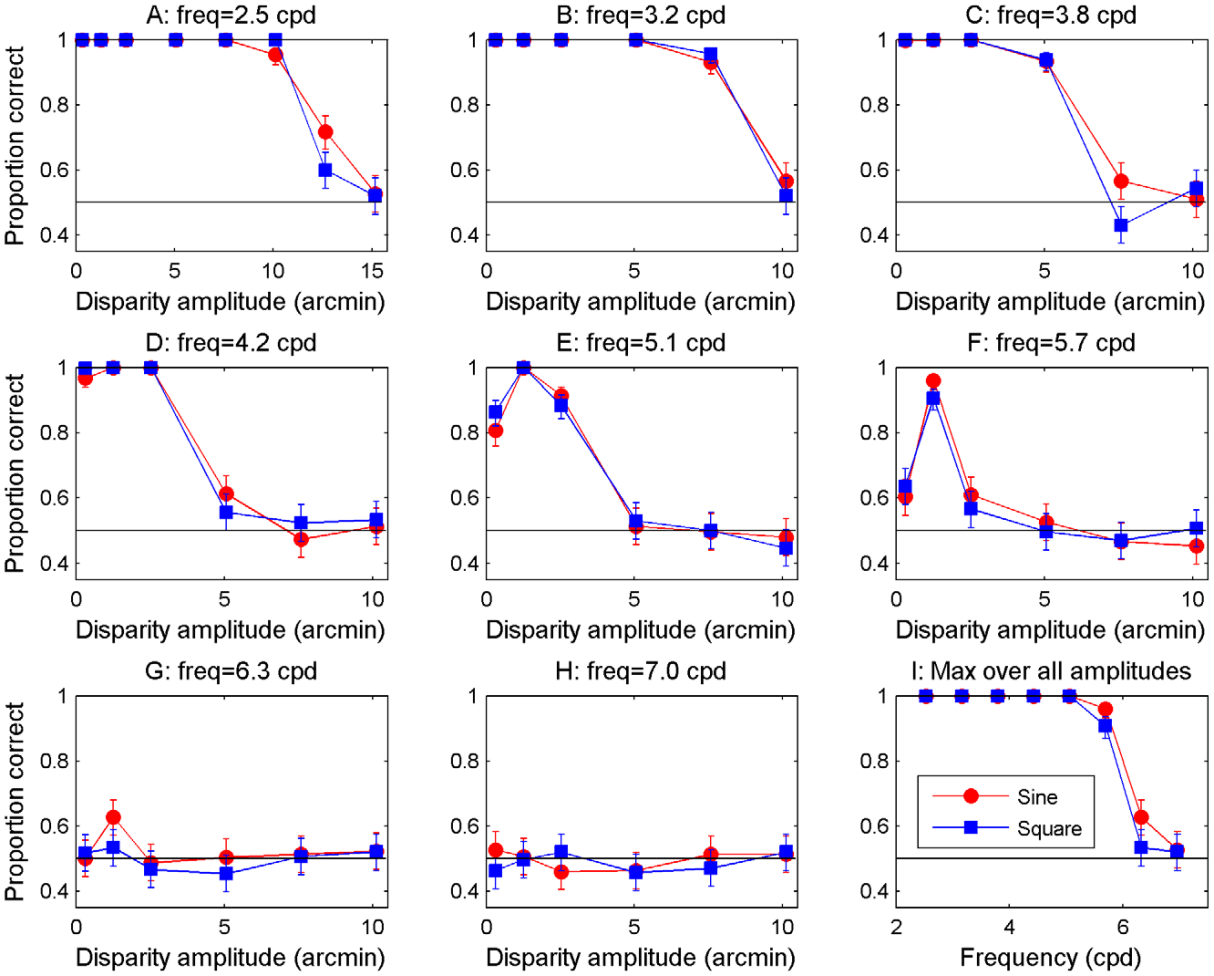
Model results with the linear size-disparity correlation. Model performance on the grating detection task as a function of amplitude and frequency. The last plot (I) shows the maximum performance over all amplitudes for each frequency. This is for the model with the template matching decision model with known frequency and a linear size-disparity relationship (Equation 3).

The results in Figure 5 assumed a quadratic relationship between a correlator's window-size and its preferred disparity. The psychophysical data suggests there may be noticeable inter-subject variation in the relationship between spatial scale and disparity correlation, with Smallman & McLeod's two subjects showing linear and quadratic relationships respectively. However, all our subjects showed near-identical performance on sine- and square-wave gratings [10]. We therefore wanted to check that the precise form assumed for the size-disparity correlation was not critical for our results. To this end, we also tested the model with a linear size/disparity correlation (Equation 3). The results (Figure 6) are similar to those obtained with the second order polynomial size/disparity correlation (Equation 2), and in particular the key result holds: differences between the sine-wave and square-wave results remain negligible. This suggests that several different forms of the size/disparity correlation may be consistent with the human data in our previous paper [10].

### Model with size-disparity correlation explains disparity gradient limit for sine and square-wave gratings

**Figure 7:**
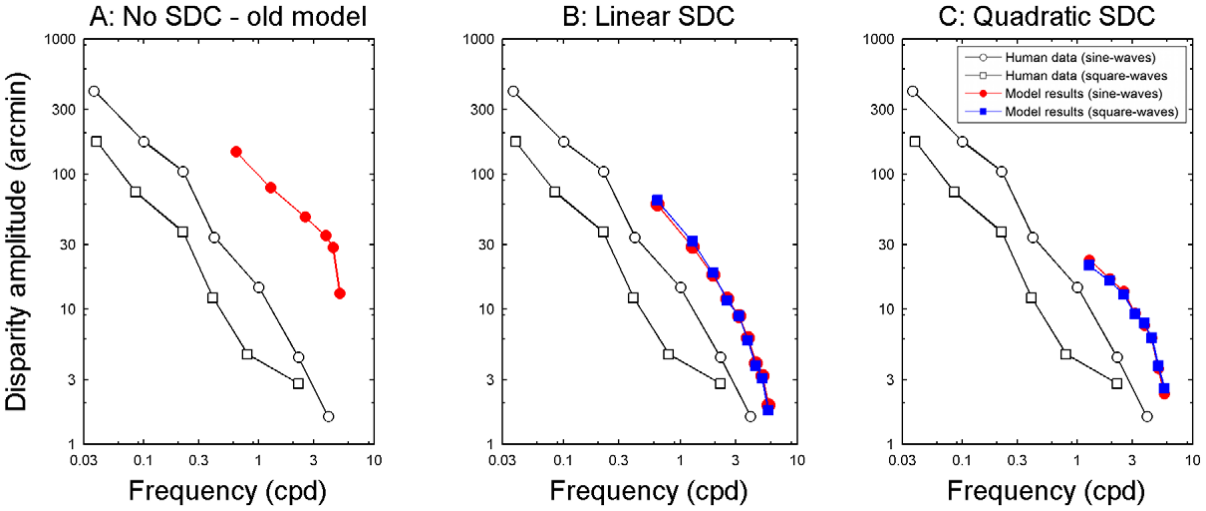
Comparison between model results and human data on the frequency dependence of the upper depth limit. The maximum amplitude at which sine- and square-wave disparity gratings can be detected with >80% accuracy, as a function of frequency. The black squares and circles show human data for square- and sine-waves replotted from Tyler [4]. The red circles show model results on sine-waves and the blue squares show model results on square-waves. A: Results with the old constant window-size model. No square-wave results are shown because the constant window-size model does not have an upper depth limit for square-waves. B: Model results using a linear size/disparity correlation in the encoding population (Equation 3). C: Model results using the same decision model but a quadratic size/disparity correlation (Equation 2).

Many previous studies have suggested that human depth perception is limited in the disparity gradients it can detect [4], [6], [8], [15], [22], [23]. For example, Tyler found that, for sinusoidal disparity gratings, the highest disparity amplitude which can be perceived is inversely proportional to grating frequency (i.e. lies on a line with a slope of minus one in log-log coordinates [4]; black symbols in Figure 7), as if perception is limited by the maximum gradient present in the grating. This observation does not require a size-disparity correlation; for example, Filippini & Banks [8] successfully reproduced it with their local cross-correlation model which incorporates no relationship between size and disparity tuning of detectors (Figure 7A). However, Tyler also found the same relationship between upper depth limit and frequency in square-wave disparity gratings. He argued that this does imply a size-disparity correlation. No computational model has yet reproduced this observation. To examine this, we re-ran our simulations using a larger range of correlation detectors, including detectors tuned to disparities up to 140 arc min. This enabled us to probe the model's upper depth limit even at frequencies <1 cpd, where performance remains perfect up to tens of arc min.

The coloured symbols in Figure 7 shows the upper limit of disparity amplitude, defined as the maximum amplitude for which performance exceeds 80% on our grating detection task, as a function of grating frequency. For comparison, Tyler's results are replotted in black. Figure 7A shows our results with the original, constant window-size model. For sinusoidal disparity gratings, the upper limit falls as a power-law with frequency, replicating the finding of Filippini & Banks. However, the model fails completely for square-wave gratings. No results are shown since the model has no upper depth limit for square-wave gratings; performance remains optimal at all amplitudes up to Panum's fusional limit, with no trade-off between upper depth limit and frequency. This is inconsistent with Tyler's data showing that, for human subjects, the upper depth limit for square-waves falls with increasing frequency in the same way as it does for sine-waves [4], as well as with our own data [10].

Figure 7B shows the results of the new model using a linear size/disparity correlation (Equation 3). For both square-wave and sine-wave gratings, the upper depth limit is inversely proportional to frequency, in agreement with the human data. However, in the model results the sine- and square-wave curves overlap almost perfectly while they are offset by a constant amount in Tyler's data. Tyler's data were obtained using a different stimulus, line stereograms rather than random dot stereograms, and while similar results have also been obtained with random dot stereograms for sine-waves [3], to our best knowledge the frequency dependence of the upper depth limit for square-waves has only been measured with line stereograms, making it hard to say whether this difference reflects a real problem with the model or if it is just a consequence of using a different stimulus. In the human data in our previous paper [10], some subjects seem to show a difference in the same direction as Tyler, though smaller, while others show almost no difference. But our paper only looked at high frequencies and the experiments were not designed specifically to test the upper disparity limit. Clearly, more data on the upper disparity limit for sine- vs. square-wave disparity gratings in random dot stereograms would be needed to test whether the lack of an offset between the sine- and square-wave results reflects a remaining problem with the model.

Figure 7C shows the results of the new model using a quadratic size/disparity correlation (Equation 2). The results for sine-waves and square-waves are again very similar, but now the upper depth limit rises less steeply as frequency is reduced, or put another way, the highest frequency detectable for a given amplitude decreases at an accelerating rate as the amplitude increases.

## Discussion

The idea of primary visual cortex as a cyclopean retina goes back to Julesz [24]. Recently, the suggestion has emerged that certain key aspects of human depth perception, notably the low spatial resolution for stereo depth, are set by the initial encoding of disparity in primary visual cortex (V1). This suggestion has been quantified with models closely based on known physiology, in which disparity is encoded via a local cross-correlation of the two eye's images, within a finite window [6], [8], [23]. In a previous study [10], we identified a problem with the current implementation of this model. The model predicts a difference between the detectability of sine- vs square-wave gratings which is not observed in humans. The model predicts that, for sine-wave gratings, performance should decline from its peak value as disparity amplitude increases, while for square-wave gratings, performance should remain high. In humans, performance declines for both types of gratings. Clearly, the model needed to be altered to account for these observations.

This then raised the question of what sort of modifications were needed. Potentially, the discrepancies might reflect the model's failure to include more elaborate disparity processing in extra-striate cortex. For example, some extra-striate areas contain neurons that are tuned to disparity-defined edges, slant and curvature [11]–[14], [25], [26]. These are not included in the model. If such extra-striate mechanisms turn out to play a critical role in setting spatial stereoresolution, this would undermine the claim that stereoresolution is limited by the initial encoding of disparity performed in striate cortex. However, current models also ignore many known features of primary visual cortex, partly for practical reasons (simulation runtimes rapidly become unmanageable if one attempts to include all known variations) and partly for theoretical ones (insight is gained by abstracting out the key features which are responsible for a particular behaviour). Thus, it seemed to us that the first line of inquiry should be to explore whether a more realistic representation of the initial disparity encoding stage could reconcile the model with human behaviour.

One obvious property neglected by the current model is the tuning of neurons in early visual cortex to luminance spatial frequency and orientation. Rather, as we have shown in the first section of the Results, the model's idealised, isotropic cross-correlators represents the combined output of many such tuned neurons (as for example in [21]). For the broad-band random-dot patterns used here, we believe that this simplification is adequate, and unlikely to affect the model's performance on the particular tasks under consideration. We therefore chose to address, instead, another property ignored by current models, namely the size/disparity correlation. Much previous psychophysical work has indicated a correlation between the spatial scales over which disparity is extracted, and the amplitude of the disparity itself [4], [15]–[17]. Physiologically, this implies that a population of neurons tuned to low spatial frequencies would encode disparities over a larger range than a population with tuned to high spatial frequencies. There is some physiological evidence supporting this [18]. In the correlation model, spatial frequencies are not explicitly represented, but the integration implicitly includes all spatial frequencies with the same weighting (a limitation we discuss further below). Thus it is difficult to incorporate a relationship between disparity and spatial frequency tuning. However, it is easy to incorporate a relationship between disparity and receptive field size. We believed that such a size/disparity correlation could potentially account for the poor human performance on square-wave gratings. Our reasoning was that square-wave gratings present a greater magnitude of disparity, averaged across a cycle, than sine-wave gratings of the same amplitude. Thus, their disparity should be encoded by cross-correlators with larger average window-size than sine-wave gratings. When the window-size associated with the largest disparity in the grating is comparable to or larger than half the spatial period of the grating this effect will tend to reduce performance on square-wave gratings relative to sines, although the piecewise-frontoparallel nature of square-wave gratings will tend to enhance performance relative to sines. We wondered whether, with an appropriate relationship between window-size and disparity magnitude, these two effects could cancel out and thus account for the very similar human performance on both types of gratings.

In this paper, we have shown that our intuition was correct. Introducing a size/disparity correlation into the initial stage of disparity encoding, such that larger disparities are detected using larger correlation windows, solves both of the problems we identified with earlier version of the model. We have investigated various decision models, and shown that the model's performance does not depend critically on the particular decision model used. Rather, it reflects the information available at the initial encoding stage, for the reasons we now discuss.

### How it works: why a size-disparity correlation reconciles the model with human performance on square-wave gratings

Correlation-based models are built of disparity detectors which respond maximally, i.e. with correlation output 1, to uniform stimulus disparity at their preferred value. Stimulus disparities away from the preferred value cause a decline in the reported correlation output. In this type of model, the rate of the decline is ultimately limited by the point-spread function of the eye, with an SD of around 2 arcmin.

**Figure 8:**
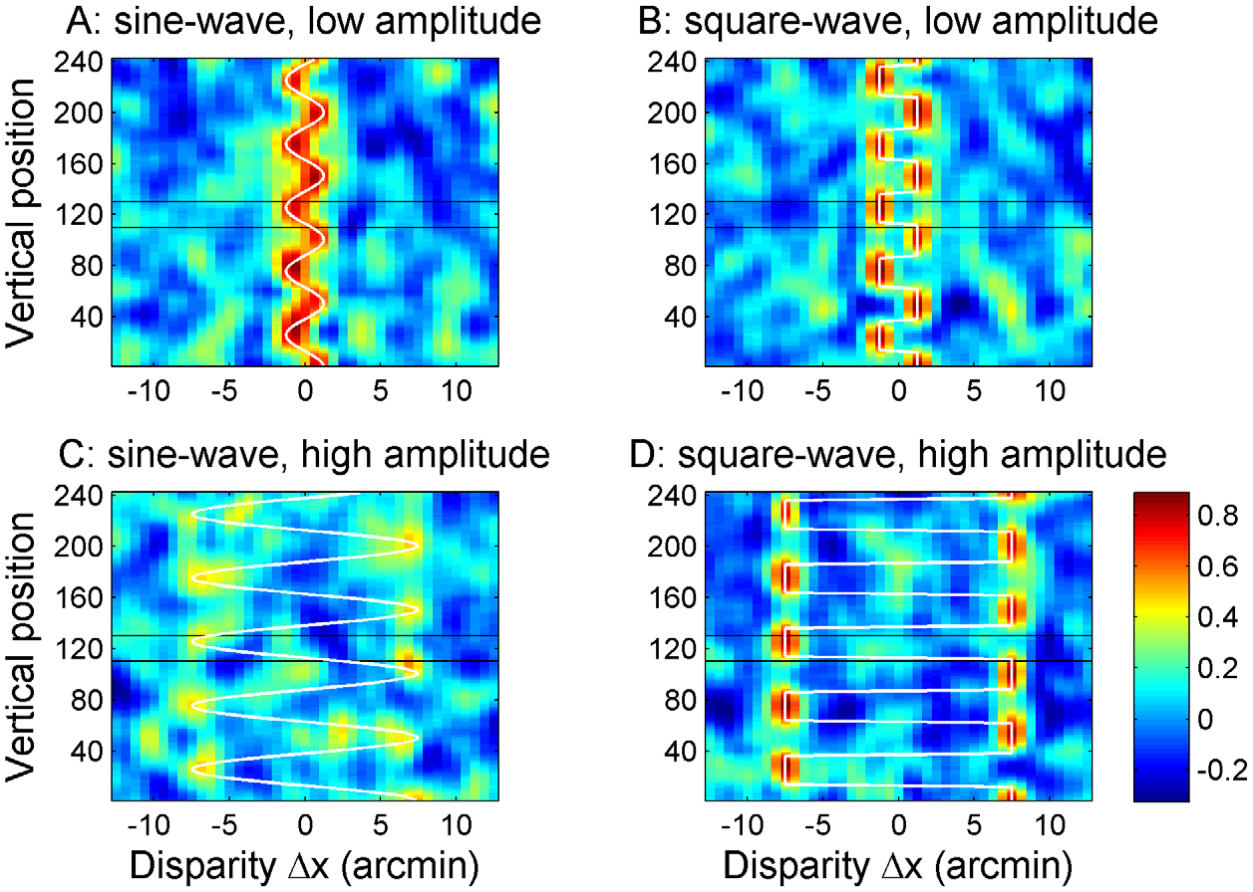
Examples of correlator output for low and high amplitude disparity gratings with the old fixed window-size model. Examples of output from the cross-correlator for the old model at a frequency of 3.8 cpd. The top row shows output for a sine-wave (A) and a square-wave (B) with low amplitude (4 pixels = 1.3 arcmin) while the bottom row shows output for a sine-wave (C) and a square-wave (D) with high amplitude (24 pixels = 7.6 arcmin). Notice that the quality of the correlator output remains high for the high amplitude square-wave (D) while only the regions close to the peaks are visible in the output for the high amplitude sine-wave (C).

In the old, fixed-window-size model, the quality of the correlator output declines with increasing amplitude for the sine-waves, but not for the square-waves. Figure 8 shows examples of the old model's correlator output for sine- and square-waves with low and high amplitude, for a frequency of 3.8 cpd. The white lines show which disparity was actually presented at each vertical position. The black lines show the extent of a correlation window, defined as the 1SD contour of the Gaussian. For the low amplitude gratings (Figure 8AB), the correlator output is of high quality for both waveforms. It is maximal at the front and back surfaces of each waveform, where the range of stimulus disparities within the correlation window is smallest. In this example, the grating half-period is 7 arcmin, so for the square-wave, detectors positioned at the center of the grating's front and back surfaces experience uniform stimulus disparity everywhere within their 6-arcmin correlation window. Detectors tuned to the stimulus disparity will therefore respond close to their maximum possible value of 1. Even at the edges of the square-wave the window will only experience two disparities, each covering half the window, allowing the correlation to be relatively high (close to 0.5) for detectors tuned to either of these two disparities. For the sine-wave, the stimulus disparity is constantly varying. However, detectors positioned at the peak and trough of the gratings experience only a small (0.8-arcmin) range in disparity within their correlation window, so the response is still high at the front and back surfaces. Even detectors at the centre of the grating (zero disparity) experience a range of only 2.4-arcmin disparity, and so give a clear, though reduced, response.

For the high-amplitude sine-wave grating, Figure 8C, the situation is very different. Detectors at the centre of the grating now experience a 14-arcmin range of stimulus disparities. There is thus almost no visible response to the slanting regions of the grating which can be distinguished from chance responses to particular random dot patterns within the stimulus. Detectors centred on the peaks and troughs of the sine-wave experience a lower disparity range of 4.8 arcmin, and periodic blobs of higher activation are still just visible here. Thus overall, the high-amplitude sine-wave grating is barely visible in the correlator output. For the high-amplitude square-wave, Figure 8D, little is changed compared to the low-amplitude case, Figure 8B. Detectors in the center of the grating's front and back surfaces still experience uniform disparity, and so their response is undiminished. Detectors at the edges of the square-waves still only experience two disparities. That these are now further apart makes no difference: each disparity is still seen by half the window allowing correlations of about 0.5 even close to the edges. This is why the old model performed so much better with high-amplitude square-waves than with sines (Figure 2, bottom row).

**Figure 9:**
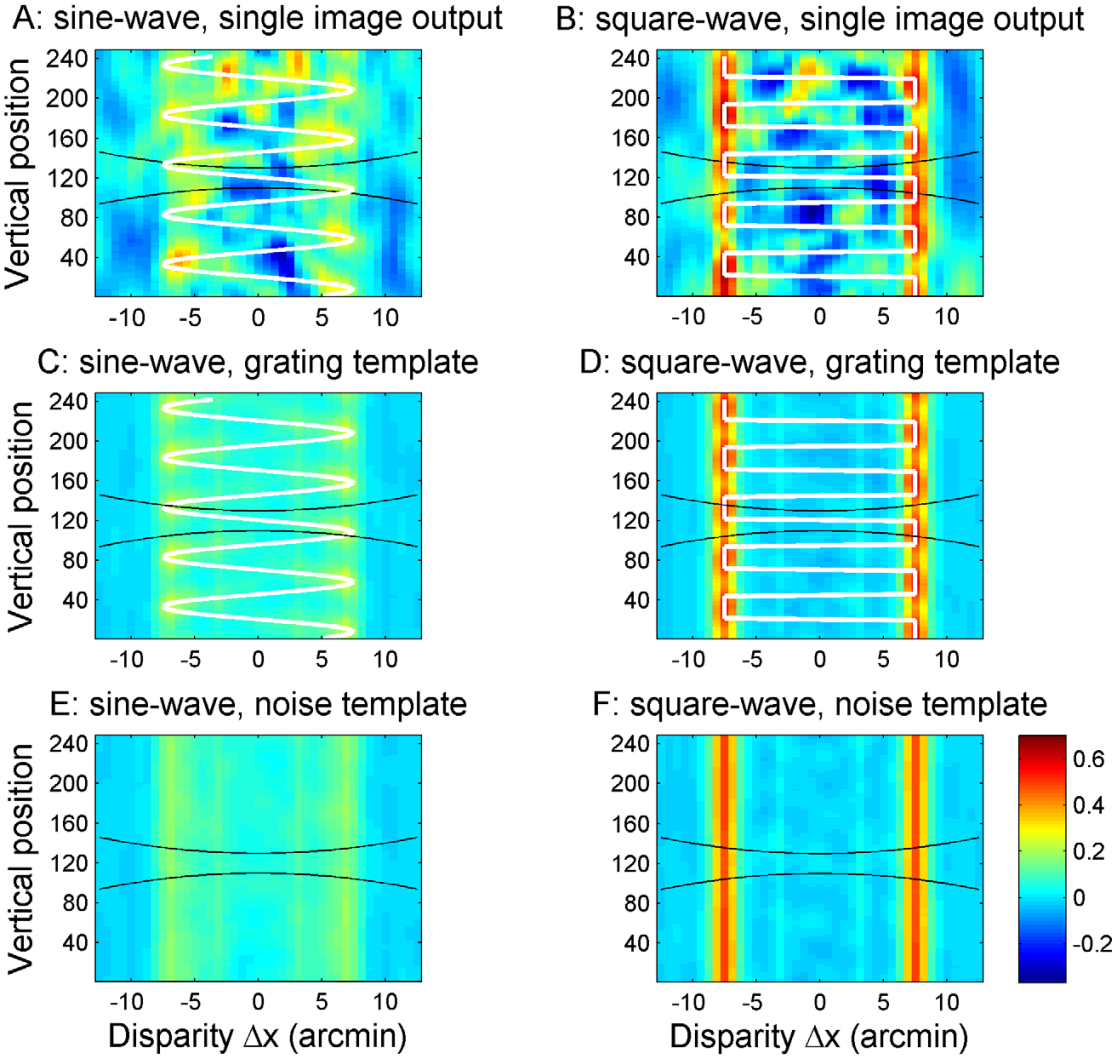
Comparison between single image correlator output, grating templates and noise templates at a high spatial frequency. The top row shows examples of output from the cross-correlator for the new model at a frequency of 3.8 cpd for sine-waves (A) and square-waves (B). The middle row shows grating templates at the same frequency for sine-waves (C) and square-waves (D). The bottom row shows noise templates for sine-waves (E) and square-waves (F). The correlator output matches the grating templates better than the noise templates.

How does the size/disparity correlation change things? Figure 9AB shows correlator output for our new model, for high amplitude sine- and square-waves at 3.8 cpd, the same frequency that was used in Figure 8. For the low amplitude gratings, the correlator output remains almost exactly the same as shown in Figure 8AB, since the window-size remains close to that used in the fixed- window-size model. For high-amplitude gratings on the other hand, considerably larger windows will be used to detect the large disparities, as indicated by the black lines. For sine-wave gratings, this has relatively little effect. Detectors at the peaks and troughs of the grating now have a window-size of 2σ = 10 arcmin. The range of disparity they experience within their correlation window is therefore larger, at 10.7 arcmin as compared to 4.8 in Figure 8C. The correlation output in Figure 9A is therefore somewhat reduced compared to the old model, Figure 8C (note slightly different colorscale), but the grating is still visible in the periodic “blobs” of higher correlation. For the square-wave, on the other hand, the increase in window-size has a more serious effect. The window now exceeds the grating half-period, meaning that correlation detectors at the middle of the front or back surfaces no longer sample only their preferred disparity, but also some disparities 15 arcmin away from their preferred value. Detectors at different vertical positions now vary only in the proportion of dots which are at their preferred disparity. Accordingly, not only are the “blobs” marking each front and back surface now lower in amplitude, but critically, they are no longer separated by clear regions of low activation (compare Figure 9B vs Figure 8D).

This is very damaging to the model's performance. Recall that, in order to assess spatial resolution, observers were asked to discriminate stimuli in which disparities were arranged as a periodic function of position (gratings) from those in which the same disparities were scattered at random (noise). Figure 9CDEF shows the mean correlator output for both types of stimuli: that is, the grating templates for this frequency and amplitude (Figure 9CD), and the noise templates for this amplitude (Figure 9EF). The model's task, then, is essentially to decide whether the output to a given stimulus, Figure 9A and B, is a better match to the grating templates in Figure 9CD or to the noise templates in Figure 9EF. These are distinguished only by their periodicity.

For the square-wave grating, the periodicity was perfectly clear with the fixed-window-size model (Figure 8CD), and is much less obvious with the size-disparity correlation model (Figure 9AB), thanks to the larger window sizes at the relevant disparities. In the new model, both the sine-wave and the square-wave output is now hard to distinguish from the noise patterns. This is why all our decision models gave similar results for both square-wave and sine-wave gratings. For the frequency and amplitude used in this example, the template matching decision model with known frequency performed at about 80% correct for both.

### Initial encoding not decision model is critical

Although we have concentrated on the template-matching decision model when explaining why the size-disparity correlation has the effect it does, qualitatively similar results were obtained from all four decision models examined (see Text S1 and Text S2). We conclude that stereoresolution is limited by the initial encoding of disparity, not by the particular read-out we have adopted. Similar conclusions were reached by Banks [6], [8] and Harris et al [27].

### Size-disparity correlation and the disparity gradient limit

Previous studies have suggested that our perception of depth patterns containing a large range of disparities may be limited by disparity gradient rather than the large disparities as such [6], [8], [15], [22], [23]. In particular a study by Tyler [4] found that the maximum depth limit, the disparity amplitude at which depth differences are no longer perceived in sinusoidal and square-wave disparity gratings, depends on corrugation frequency in a way that approximately corresponds to a straight line with slope −1 in log-log coordinates. Banks et al. [8] had previously shown that a constant window size local cross-correlation model performed in a qualitatively similar way when tested with sinusoidal disparity gratings. Here, we have replicated this finding and shown that when a size/disparity correlation is incorporated into the model it performs in the same way for square-wave disparity gratings, consistent with Tyler's results. The model achieves this despite lacking any sensors tuned to non-zero disparity gradients. Banks et al. suggested that the disparity gradient limit was a by-product of using local cross-correlation to estimate disparity [6],[8]. However, as Tyler [4] recognized, this alone cannot explain why the frequency dependence of the upper depth limit exists for square-waves as well as for sine-wave gratings. We have found that incorporating a size/disparity correlation into a correlation-based model makes it perform consistently for random-dot patterns depicting both square-wave and sine-wave disparity gratings. This supports Tyler's conclusion [4] that the disparity gradient limit reflects a size/disparity correlation, rather than being solely a by-product of local cross-correlation.

### Relationship to previous models

Models of stereopsis based on cross-correlation of local patches of the two eyes' images have a long history [23], [28]–[30]. They are widely used in computer vision as a fast and relatively reliable approach of achieving stereo correspondence. They have often been used to model human vision [6], [8], [27], [31]. Local cross-correlation is closely related to the “stereo energy” computation performed by cells in primary visual cortex [32]–[35], although cells spectrally filter the local image patches before cross-correlating them. Models based on stereo energy units have also been used as models of human vision [19], [21], [35]–[38]. All these implementations have recognized that useful disparity estimates require the outputs of many stereo energy units to be combined in some way. For example, models have estimated disparity by combining the outputs of stereo energy units with different spatial locations [35], [39], or different spatial frequencies and/or orientations [21], [36], [40]. As we show in this paper, combining stereo energy units tuned to many different spatial frequencies and orientations can produce something which is formally identical to local cross-correlation of the unfiltered image.

Stereo energy units based on phase disparity [32], [41] naturally incorporate a size-disparity correlation. In this type of disparity encoding, the unit's preferred disparity Δx is roughly Δφ/2πf, where Δφ is its preferred phase and *f* its preferred spatial frequency. If the largest phase disparity and bandwidth are the same for all spatial scales, then the largest preferred disparity is inversely proportional to frequency and thus proportional to size. Tsai & Victor [19] used stereo energy units with phase disparity which therefore incorporated a size-disparity correlation. They showed that this model, with template-matching, was able to account for stereoacuity as a function of frequency in sine-wave luminance gratings (NB these are luminance gratings at a constant depth, not random-dot patterns depicting sinusoidal depth modulation as in the present paper). Our model uses position disparity, in which size-disparity correlation does not arise naturally, but has been built in by design. This leads to an important difference between the two implementations. Our size-disparity correlation links disparity to the size of the window across which disparities are sought, but not to spatial frequency. Our correlation-based model includes information from all spatial frequencies, independent of window-size. Thus, the meaning of “size-disparity correlation” is somewhat different in the two cases.

### Limitations of the model

Our model suffers from many limitations, most of which were forced on us by the difficulty of running simulations with large numbers of neurons. Most previous studies have either used stimuli with a uniform disparity profile, meaning that it suffices to model neurons at only one location in the visual field [19]–[21], or have modelled neurons at several locations but with only one spatial frequency and orientation [37]. In order for the model to detect gratings that vary in depth, we needed to compute responses in many locations in the visual field. It would have been very costly also to model the responses of stereo energy units tuned to many different spatial frequencies and orientations. We therefore used the cross-correlation technique [6], [8], [27], [37] as a convenient short-cut to approximate the responses of many stereo energy units tuned to all possible frequencies and orientations.

Our analysis showing how local cross-correlation can be implemented exactly by stereo energy units is clearly idealized. Most notably, we integrated the response over all spatial frequencies, while keeping the receptive field size constant. Extending the integration to infinite spatial frequency is obviously unrealistic, although in practice will not greatly affect the results, since unrealistically high spatial frequencies will be removed from the images by the optical blurring and pre-processing. Keeping the receptive field size constant is a more serious limitation. Of course, primary visual cortex contains cells with a range of receptive field sizes. We have included only one window-size (receptive field size) at each preferred disparity. Once again, this was for reasons of computational economy. We regard the window-size within our model as representing the smallest receptive field sizes which contribute significantly to disparity detection. Ideally, we would have included a range of window-sizes at every disparity, with the smallest window-size at each disparity increasing as a function of disparity. However, since stereoresolution is limited by the smallest windows present, we would not expect this to alter our results substantially.

Keeping the receptive field size constant corresponds to postulating that bandwidth declines with spatial frequency, as it does in the macaque [42]. Assuming Gabor receptive fields, a Gaussian envelope with standard deviation 3 arcmin implies a bandwidth of 0.5 octaves at 15 cpd; at 5 cpd the bandwidth ranges from 1.5 octaves (sine phase) to 2.0 octaves (cosine phase), while at 0.5 cpd the bandwidth is 1.8 octaves for sine phase (cosine-phase cells are low-pass). These values are consistent with those reported in macaque [42]. At a given frequency, the bandwidth will be narrower for large RFs than for small ones.

As mentioned in the previous section, our correlation-based model includes information from all spatial frequencies, independent of window-size. This is a consequence of the mathematical trick we have used to integrate over frequencies. In fact, several lines of evidence suggest that larger disparities are detected predominantly by mechanisms tuned to lower spatial frequencies in the luminance domain [16], [43], [44]. Thus, it would be more realistic to include a weight term in the integration over luminance spatial frequency, weighting the integral towards lower frequencies at the larger disparities/window-sizes, and towards higher frequencies at the smaller disparities/window-sizes.

We have not included any neuronal noise within our model, nor have we attempted to reproduce human stereoacuity for gratings, i.e. the smallest disparity amplitude detectable at each frequency. In principle, it would be simple to add this. Stereoacuity is limited by the spacing of disparity detectors, and by neuronal and stimulus-dependent noise (random correlations between non-corresponding parts of the dot pattern, for example). However, stereoacuity is also clearly limited by processing in higher cortical areas and not solely by the information available in V1 [45], [46]. This means that the model's assumptions about extra-striate processing would probably play a much more critical role in reproducing stereoacuity data than they have done here in reproducing stereoresolution.

We have only modeled the detection of horizontally-oriented disparity gratings. Humans find these easier to detect than vertically-oriented gratings [7], [47]–[49]. It is currently unclear what model features would be required to match this feature of stereo vision. However, a clue may be that the disparity tuning surfaces of real cortical neurons are extended horizontally and are relatively narrow vertically [50]. In any stereo algorithm, the choice of window-size represents a trade-off between resolution and accuracy. Large windows collect support over a wider region of the image, enabling greater accuracy and robustness against false matches. However, they also lose the ability to track rapid changes in depth. For this reason, disparity steps are detected most accurately by windows which are elongated parallel to the edge and narrow orthogonal to the edge [23]. Thus, the horizontally-elongated disparity tuning surfaces of real neurons would be expected to give greater sensitivity to changes in depth along a vertical direction in the image, as observed in humans. Further modelling work is required to examine whether models which incorporate this known anisotropy in V1 neurons can reproduce the anisotropy in human depth perception.

A great deal is now known about how disparity is encoded within V1. Much less is known about how this activity is read out in higher areas to result in depth perception and judgments on tasks such as our grating detection [51]. Thus, our model is necessarily much more speculative here. Is it realistic to assume that our brains have access to “templates” representing the expected V1 output for different stimuli? Physiologically, these templates could be represented as the synaptic weights between V1 and “grating detector” units in a higher visual area (see [20] for a more detailed account). While neurons specifically tuned for disparity gratings have not been reported, “grating detector” units would also respond preferentially to disparity curvature and slant, and such neurons are known to exist in areas IT and MT [11], [12]. Alternatively, such neurons might be constructed as required. In areas such as LIP, neurons quickly adapt their responses to the particular task requirements at hand [52]. In this view, participants may be able to construct adequate templates simply from the few disparity gratings they are shown as demonstration stimuli.

### Conclusions

Local cross-correlation within a fixed window has been postulated as a model of human stereo vision. This model accounts for stereoresolution when depth is modulated sinusoidally, but gives incorrect predictions for square-waves. We have shown that introducing a size/disparity correlation, such that larger disparities are detected within coarser windows, reconciles the local cross-correlation model with human stereoresolution on both square- and sine-wave disparity gratings. This supports the original conclusion of Banks et al. [6] that the limit on spatial stereoresolution is set by the smallest receptive field size of V1 neurons, which respond best to locally frontoparallel surfaces [6], [8]. There is thus no need to invoke further limits imposed by cells in extrastriate cortex tuned to more complicated aspects of disparity such as slant and curvature. Such cells can be created by combining the outputs of V1 neurons with different preferred disparities, but in this view, they inherit a fundamental limit on stereoresolution, set in primary visual cortex.

## Supporting Information

Supporting Text S1 Contains model results using a decision model that was not given the frequency of the disparity gratings.
(PDF)

Supporting Text S2 Contains model results using a decision model based on autocorrelation.
(PDF)

Supporting Text S3 Contains a detailed calculation of the integral over the binocular energy model term used in the discussion of how local cross-correlation can be obtained from energy-model units.
(PDF)
