# Supplementary material for: Spatial Stereoresolution for Depth Corrugations May Be Set in Primary Visual Cortex
Source: PLoS Comput Biol. 2011 Aug 18;7(8):e1002142. doi: 10.1371/journal.pcbi.1002142 (PMC3158043; doi:10.1371/journal.pcbi.1002142)
Supplement: Supporting Text S1 — Contains model results using a decision model that was not given the frequency of the disparity gratings. (PDF) [file pcbi.1002142.s001.pdf]

Text S1: Decision model using template matching with unknown frequency

This method was as for that in the main body of the paper, except that $M_{grating}$ was calculated for templates of all frequencies, including two frequencies (1.9 cycles/degree and 7.6 cycles/degree) for which no results are shown (because the model performed either perfectly or at chance), not just the 432 with the correct stimulus frequency. The results are shown in Figure S1-1.

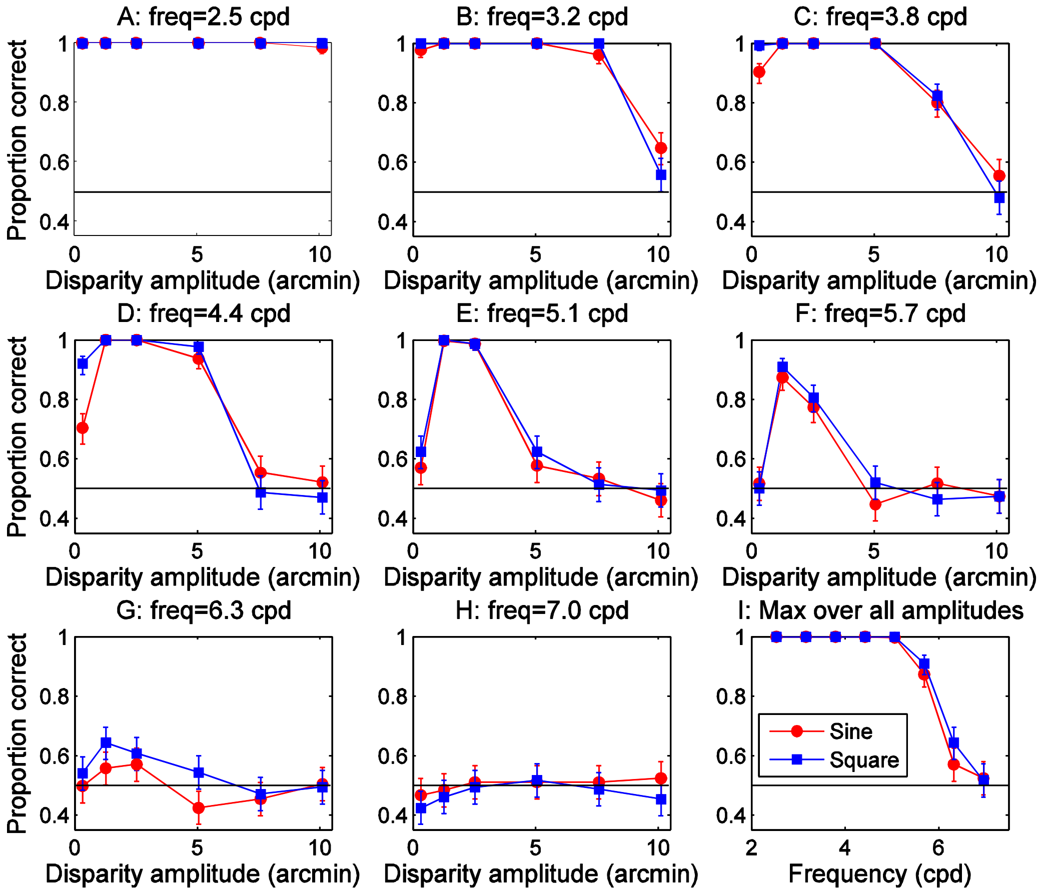

Figure S1-1: Model performance on the grating detection task as a function of amplitude and frequency. The boxed plot (I) shows the maximum performance over all amplitudes for each frequency. This is for the model with the template matching decision model with unknown frequency and a quadratic size-disparity relationship (Equation 2 in the main document).
