# Supplementary material for: Spatial Stereoresolution for Depth Corrugations May Be Set in Primary Visual Cortex
Source: PLoS Comput Biol. 2011 Aug 18;7(8):e1002142. doi: 10.1371/journal.pcbi.1002142 (PMC3158043; doi:10.1371/journal.pcbi.1002142)
Supplement: Supporting Text S2 — Contains model results using a decision model based on autocorrelation. (PDF) [file pcbi.1002142.s002.pdf]

Text S2: Decision model using Autocorrelation

Here we show results from a decision model which does not use template-matching at all, but detects the grating from the autocorrelation of the disparity map. In this decision model, we start by estimating disparity at each point on a vertical line down the image. We do this by finding the peak correlation on each row of the correlation images that were the output from the cross-correlator. The disparity at which the peak correlation was found at each row was recorded as an estimate of the horizontal disparity at the corresponding vertical position:

$$\Delta x_{est}(y) = \arg\max(C(y, \Delta x))$$

Equation 1

The result was a curve of estimated disparity as a function of vertical position (see Figure S2-1AB). The next step was to calculate the autocorrelation of this curve as:

$$a_n = \frac{\sum_{i=1}^{N-n} (\Delta x_{est}(y_i) - \mu)(\Delta x_{est}(y_{i+n}) - \mu)}{((N-n) * \sigma^2)}$$

Equation 2

where μ is the mean and σ is the standard deviation of Δx_{est}, the sum is over all vertical positions *i* and n is the lag. Figure S2-1CD shows two examples of auto-correlograms. The last step was to fit a sine-wave and a triangular wave, the auto-correlation function of a square-wave, with the same frequency used in the stimulus to the auto-correlogram and record the r^2-value of the best fit. Only the frequency of the gratings was given to the model. The amplitude was acquired by choosing the amplitude which gave the best fit and the model did not need to know the phase since the autocorrelation function is largely independent of phase. Letting the model know the frequency of the gratings was motivated because we had kept the frequency constant in each block of trials in the psychophysics experiments. The decision on which of the two image pairs given to the model in any trial contained the grating was then made by choosing the image pair which gave the highest r^2-value.

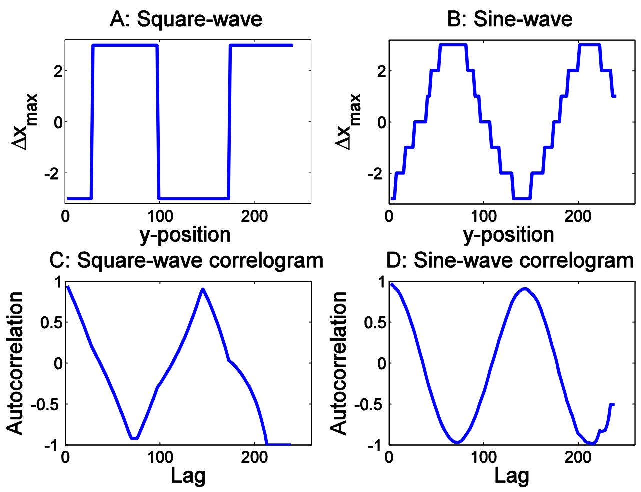

Figure S2-1: Examples of estimated disparity curves and their autocorrelograms for one square-wave and one sine-wave both with a frequency of 1.3 cpd. A Gaussian window with $2*\sigma = 6+0.063*(\Delta x)^2$ arcmin was used. The estimated disparity curve for the sine-grating is quantized because the model only included detectors tuned to integer disparities (in pixels).

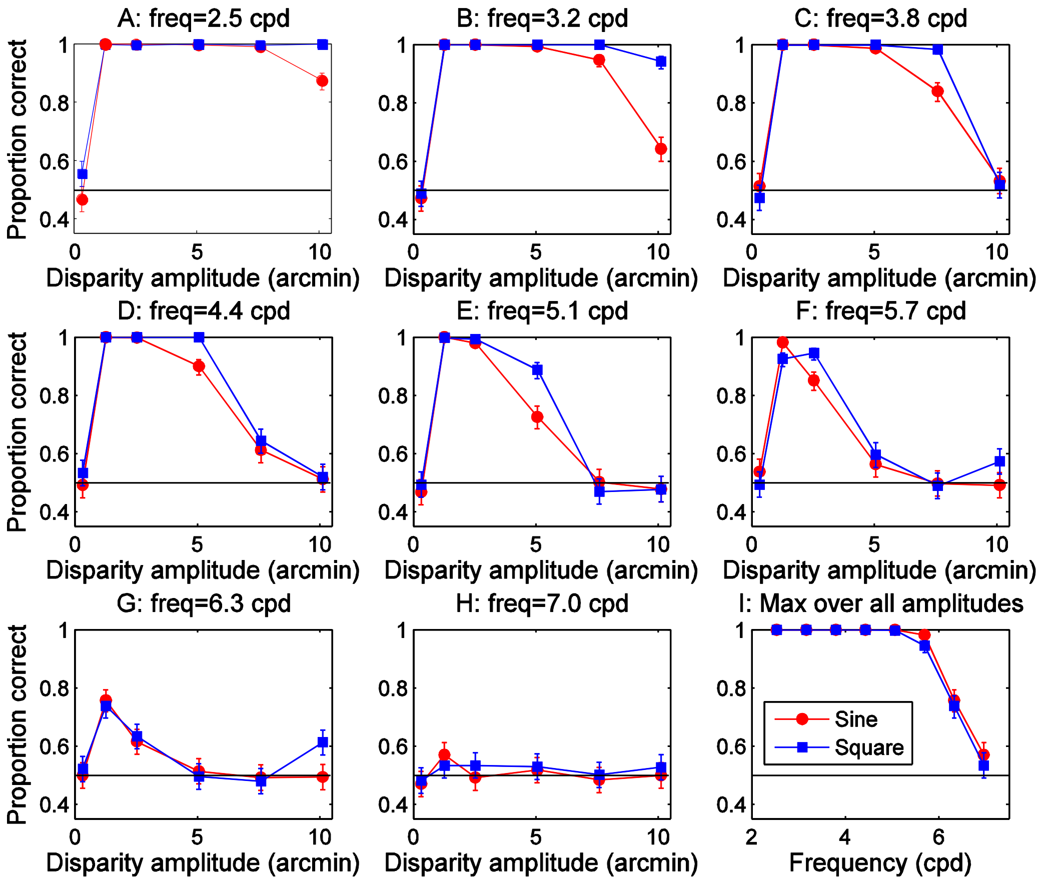

Figure S2-2: Model performance on the grating detection task as a function of amplitude and frequency. The boxed plot (I) shows the maximum performance over all amplitudes for each frequency. This is for the model with the autocorrelation based decision model and a quadratic size-disparity relationship (Equation 2 in the main document).
