# Supplementary material for: Spatial Stereoresolution for Depth Corrugations May Be Set in Primary Visual Cortex
Source: PLoS Comput Biol. 2011 Aug 18;7(8):e1002142. doi: 10.1371/journal.pcbi.1002142 (PMC3158043; doi:10.1371/journal.pcbi.1002142)
Supplement: Supporting Text S3 — Contains a detailed calculation of the integral over the binocular energy model term used in the discussion of how local cross-correlation can be obtained from energy-model units. (PDF) [file pcbi.1002142.s003.pdf]

Text S3: Integrating binocular term over frequency and orientation

The binocular term in the response of a single energy-model complex cell is

$$B = 2(S_{L1}S_{R1} + S_{L2}S_{R2})$$
$$= 2\int dxdy \int dx'dy' I_L(x,y) I_R(x',y') \exp\left(-\frac{\left((x-x_L)^2+(y-y_L)^2\right)+\left((x'-x_R)^2+(y'-y_R)^2\right)}{2\sigma^2}\right)$$
$$\left[\cos(k_x x + k_y y + \phi_L)\cos(k_x x' + k_y y' + \phi_R) + \sin(k_x x + k_y y + \phi_L)\sin(k_x x' + k_y y' + \phi_R)\right]$$

This cell is tuned to a spatial frequency and orientation specified by the wavenumbers k_x and k_y, and has receptive fields centered at (x_L,y_L) and (x_R,y_R), with phases ϕ_L and ϕ_R respectively. We now compute the total response of many such cells tuned to many spatial frequencies and orientations, but all with the same receptive field centers and phases:

$$B_{\text{int}} = \int B dk_x dk_y$$
$$= 2\int dxdy \int dx'dy' I_L(x,y) I_R(x',y') \exp\left(-\frac{\left((x-x_L)^2+(y-y_L)^2\right)+\left((x'-x_R)^2+(y'-y_R)^2\right)}{2\sigma_{RF}^{\ 2}}\right)$$
$$\int dk_x dk_y \left[\cos(k_x x + k_y y + \phi_L)\cos(k_x x' + k_y y' + \phi_R) + \sin(k_x x + k_y y + \phi_L)\sin(k_x x' + k_y y' + \phi_R)\right]$$

Doing the innermost integral first, we obtain

$$\int dk_x dk_y \left\{\cos(k_x x + k_y y + \phi_L)\cos(k_x x' + k_y y' + \phi_R) + \sin(k_x x + k_y y + \phi_L)\sin(k_x x' + k_y y' + \phi_R)\right\}$$
$$= \frac{1}{4}\int dk_x dk_y \begin{Bmatrix} \left[\exp i(k_x x + k_y y + \phi_L) + \exp -i(k_x x + k_y y + \phi_L)\right]\left[\exp i(k_x x' + k_y y' + \phi_R) + \exp -i(k_x x' + k_y y' + \phi_R)\right] \\ -\left[\exp i(k_x x + k_y y + \phi_L) - \exp -i(k_x x + k_y y + \phi_L)\right]\left[\exp i(k_x x' + k_y y' + \phi_R) - \exp -i(k_x x' + k_y y' + \phi_R)\right] \end{Bmatrix}$$
$$= \frac{1}{4}\int dk_x dk_y \begin{Bmatrix} \exp i(k_x x' + k_y y' + \phi_R)\exp i(k_x x + k_y y + \phi_L) + \exp i(k_x x' + k_y y' + \phi_R)\exp -i(k_x x + k_y y + \phi_L) \\ + \exp -i(k_x x' + k_y y' + \phi_R)\exp i(k_x x + k_y y + \phi_L) + \exp -i(k_x x' + k_y y' + \phi_R)\exp -i(k_x x + k_y y + \phi_L) \\ - \exp i(k_x x' + k_y y' + \phi_R)\exp i(k_x x + k_y y + \phi_L) + \exp i(k_x x' + k_y y' + \phi_R)\exp -i(k_x x + k_y y + \phi_L) \\ + \exp -i(k_x x' + k_y y' + \phi_R)\exp i(k_x x + k_y y + \phi_L) - \exp -i(k_x x' + k_y y' + \phi_R)\exp -i(k_x x + k_y y + \phi_L) \end{Bmatrix}$$
$$= \frac{1}{2}\int dk_x dk_y \left\{\exp i(k_x x' + k_y y' + \phi_R)\exp -i(k_x x + k_y y + \phi_L) + \exp -i(k_x x' + k_y y' + \phi_R)\exp i(k_x x + k_y y + \phi_L)\right\}$$
$$= \frac{1}{2}\exp i(\phi_R - \phi_L)\int dk_x dk_y \left\{\exp i(k_x x' + k_y y')\exp -i(k_x x + k_y y)\right\}$$
$$+ \frac{1}{2}\exp i(\phi_L - \phi_R)\int dk_x dk_y \left\{\exp -i(k_x x' + k_y y')\exp i(k_x x + k_y y)\right\}$$
$$= \frac{1}{2}\exp i(\phi_R - \phi_L)\int dk_x dk_y \exp i\left(k_x(x'-x) + k_y(y'-y)\right) + \frac{1}{2}\exp i(\phi_L - \phi_R)\int dk_x dk_y \exp i\left(k_x(x-x') + k_y(y-y')\right)$$
$$= \frac{1}{2}\left(e^{i\phi_R - i\phi_L} + e^{-i\phi_R + i\phi_L}\right)\delta(x-x')\delta(y-y') = \cos(\Delta\phi)\delta(x-x')\delta(y-y')$$

where $\Delta\phi = \phi_R - \phi_L$ is the phase disparity of the cells. Using this result in the equation for the integral of B gives us:

$$B_{\text{int}} = \int B dk_x dk_y = 2\cos(\Delta\phi)\int dxdy \exp\left(-\frac{\left((x-x_L)^2+(y-y_L)^2\right)}{2\sigma^2}\right) I_L(x,y) \exp\left(-\frac{\left((x-x_R)^2+(y-y_R)^2\right)}{2\sigma^2}\right) I_R(x,y)$$
